# Cytokine Signaling in Multiple Sclerosis and Its Therapeutic Applications

**DOI:** 10.3390/medsci5040023

**Published:** 2017-10-13

**Authors:** Pushpalatha Palle, Kelly L. Monaghan, Sarah M. Milne, Edwin C.K. Wan

**Affiliations:** 1 Department of Microbiology, Immunology, and Cell Biology, West Virginia University School of Medicine, Morgantown, WV 26506, USA; pushpalatha.palle@hsc.wvu.edu (P.P.); klm0031@mix.wvu.edu (K.L.M.); sarah.milne@hsc.wvu.edu (S.M.M.); 2 Center for Basic and Translational Stroke Research and the Center for Neurodegenerative Diseases, Blanchette Rockefeller Neurosciences Institute, West Virginia University School of Medicine, Morgantown, WV 26506, USA

**Keywords:** multiple sclerosis, experimental autoimmune encephalomyelitis, cytokines, granulocyte-macrophage colony-stimulating factor

## Abstract

Multiple sclerosis (MS) is one of the most common neurological disorders in young adults. The etiology of MS is not known but it is widely accepted that it is autoimmune in nature. Disease onset is believed to be initiated by the activation of CD4^+^ T cells that target autoantigens of the central nervous system (CNS) and their infiltration into the CNS, followed by the expansion of local and infiltrated peripheral effector myeloid cells that create an inflammatory milieu within the CNS, which ultimately lead to tissue damage and demyelination. Clinical studies have shown that progression of MS correlates with the abnormal expression of certain cytokines. The use of experimental autoimmune encephalomyelitis (EAE) model further delineates the role of these cytokines in neuroinflammation and the therapeutic potential of manipulating their biological activity in vivo. In this review, we will first present an overview on cytokines that may contribute to the pathogenesis of MS or EAE, and provide successful examples and roadblock of translating data obtained from EAE to MS. We will then focus in depth on recent findings that demonstrate the pathological role of granulocyte-macrophage colony-stimulating factor (GM-CSF) in MS and EAE, and briefly discuss the potential of targeting effector myeloid cells as a treatment strategy for MS.

## 1. Introduction

Multiple sclerosis (MS) is one of the most common neurological diseases in young adults, with initial clinical signs often observed between 20 and 45 years of age [1]. It is more prevalent in females than in males, with incidence ratio of about 3:1 [2]. The exact mechanism accounting for this gender difference is unclear, but possibly due to the effect of gonadal hormones. Currently, more than two million people suffer from MS worldwide and over 400,000 people are affected in the United States [3,4]. Given the early onset and lifelong progression of the disease, MS poses significant psychological burden to the patients and their families, as well as economic burden to society [5]. Characteristics of MS pathology include plaque formation in the central nervous system (CNS) due to inflammation and demyelination, which result in clinical manifestations such as vision impairment, cognitive problems, loss of muscle coordination, weakness and fatigue, numbness, depression, bowel changes, and bladder dysfunction, depending on which area(s) of the CNS is affected [1,6,7].

## 2. Diagnosis and Clinical Courses of Multiple Sclerosis

The wide range of clinical manifestations of MS overlaps with other types of neurological disorders, such as acute disseminated encephalomyelitis and neuromyelitis optica spectrum disorder. Therefore, early and precise diagnosis is needed for proper disease management and treatments. Diagnosis of MS is heavily based on results from magnetic resonance imaging (MRI) depicting lesion(s) dissemination in space (DIS) and time (DIT) [8]. MRI is also a valuable tool for monitoring disease progression and effectiveness of treatments, based on the number of newly formed lesions [9]. In addition, paraclinical tests showing evidence of elevated immunoglobulin G (IgG) index or evidence of oligoclonal bands in the cerebrospinal fluid (CSF) further confirms certain cases of MS [10].

The clinical course of MS varies among patients and is categorized based on the frequency of attacks (relapses) and the patterns of disease progression [11]. Relapsing-remitting MS (RRMS) is the most prevalent subtype which affects ~85% of patients [1]. The clinical pattern of RRMS is marked by unpredictable relapses resulting in acute neurological disability, followed by disease remission. Patients may have partial or complete recovery during each remission [1,12]. Unfortunately, disease pattern changes over time and patients suffering from RRMS eventually develop secondary progressive MS (SPMS), characterized by slow but significant increase in disease severity without remission [13]. Primary progressive MS (PPMS) affects ~10% of MS patients, which is characterized by progressive deterioration of neurological functions from the onset of disease symptoms without relapse and remission [14,15]. Pathological and MRI features of SPMS and PPMS are indistinguishable, suggesting the lack of relapsing-remitting phase in PPMS may be due to attacks at the clinically silent regions during the early stage of the disease. Once damage accumulates in the CNS, clinical manifestations of MS become apparent [16,17].

## 3. Multiple Sclerosis as an Immune-Mediated Disease

The etiology of MS is not well-defined but likely multifactorial, involving both genetic and environmental factors. MS is generally considered an autoimmune disease, with support from several lines of evidence. Genome-wide association studies (GWAS) identified over 100 MS risk loci, many of which overlap with those identified from other autoimmune diseases [18,19]. The majority of these loci regulate or encode genes that control immune cell functions, such as human leukocyte antigen (HLA) [20]. Moreover, immune cells infiltrating from the periphery, especially T cells and myeloid cells, are often identified around the demyelinating plaques [21,22]. Importantly, although the frequency of peripheral myelin-reactive T cells is comparable between MS patients and healthy donors, those cells from the patients express higher amounts of pro-inflammatory cytokines [23], supporting the autoimmune nature of MS.

Vitamin D deficiency and Epstein-Barr virus (EBV) infection are considered the most relevant extrinsic risk factors of MS, and both of them to some extent are due to their dysregulating effects on the immune system. The bioactive form of vitamin D, i.e., 1, 25 dihydroxyvitamin D, regulates both innate and adaptive immune responses by modulating T helper (Th)-cell and B-cell differentiation, while also maintaining the tolerogenic status of dendritic cells (DCs), which suppress T-cell activation [24,25,26]. In addition, recent studies suggest that polymorphisms of the vitamin D receptor gene are associated with the incidence of MS [27,28]. MS patients often test positive in serological analysis for EBV [29,30]. EBV is a human herpesvirus capable of infecting B cells, resulting in infectious mononucleosis in adults [31]. It is not clear how EBV infection links to the pathogenesis of MS, though there is speculation that infection mediates molecular mimicry and/or bystander activation of antigen-presenting cells (APCs) and autoreactive T cells [32].

The most convincing evidence to support the immune-mediated nature of MS comes from the fact that the two most effective disease-modifying drugs currently available for RRMS, natalizumab and fingolimod, block leukocyte egress from the periphery and infiltration into the CNS [33]. Natalizumab blocks the interaction of very late activation antigen (VLA)-4 expressed on Th1 cells with the vascular cell adhesion molecule (VCAM)-1, thus preventing extravasation of these cells [34]. Fingolimod, on the other hand, down-regulates sphingosine-1-phosphate receptor 1 (S1PR1) and selectively traps the CCR7^+^ central memory T cells in the lymph nodes [35]. The relatively high efficacy (55–65% reduction in relapses) of these drugs also suggests that MS is initiated by abnormal activation of peripheral T cells [36,37].

## 4. Experimental Autoimmune Encephalomyelitis Is a Valuable Model for Multiple Sclerosis Research

Research over the past few decades has focused on identifying immune cell types and mediators involved in the pathogenesis of MS, with an ultimate goal of improving clinical outcomes. Although scientists and clinicians have learned much by analyzing the cellular and molecular profiles of CSF and peripheral blood from the patients, as well as postmortem tissue examination, the causal relationship between experimental results and disease initiation and progression is difficult to establish. Clinical trials of mechanistically well-defined drugs are one way to identify critical components contributing to the pathogenesis of MS, but these trials are often difficult to initiate without support from experimental data. Therefore, animal models play a critical role in not only dissecting the molecular pathways governing the pathogenesis of MS, but also for identifying new therapeutic agents. Experimental autoimmune encephalomyelitis (EAE) is the best animal model available to study MS [38,39]. EAE can be induced in various species but mice are most often used given the vast repertoire of genetically-modified animals [39]. Neuroinflammation, subsequent paralysis and physical disability can be induced by active immunization of mice with self-antigen derived from the CNS, including myelin basic protein (MBP), myelin oligodendrocyte glycoprotein (MOG), or proteolipid protein (PLP), emulsified in complete Freund’s adjuvant (CFA) [39]. Further studies showed that immunization with antigenic epitopes of those proteins, such as MOG_35–55_ and PLP_139–151_ peptides, results in EAE with comparable disease severity [40,41,42]. Active immunization is often supplemented with injections of pertussis toxin, which facilitate the recruitment of leukocytes to the CNS and increase permeability of the blood–brain barrier (BBB) [43]. Recently, it was reported that injection of pertussis toxin drives an early production of interleukin (IL)-1β, which is important for the priming phase of EAE [44]. Depending on the mouse strains, in addition to the choice and dosages of peptides, patterns of EAE can be manipulated from relapsing-remitting to progressive subtypes, resembling MS pathology in humans [39]. EAE can also be induced by adoptive transfer of the autoreactive CD4^+^ T cells purified from actively immunized mice, or CD4^+^ T cells isolated from the transgenic mouse strain in which the T-cell receptor (TCR) specifically recognizes MOG_35–55_ (the 2D2 mice) [45,46]. In the latter case, CD4^+^ T cells are purified from the unimmunized mice, and then skewed to different T-helper lineages in vitro before injecting these cells to the recipient mice [39]. This approach is particularly useful to dissect the distinctive roles of Th1 and Th17 cells in neuroinflammation. Indeed, EAE was one of the first disease models used to elucidate the pathogenic role of Th17 cells [47,48].

Besides the elucidation of immune cell types and molecular pathways governing the pathogenesis of neuroinflammation, EAE model has also been widely used for testing potential therapeutic agents for MS, and some of them have been successfully translated to the clinic. Glatiramer acetate (GA, Copaxone, Teva Pharmaceutical Industries Ltd., Petah Tikva, Israel), a first-generation MS drug, was first proven to effectively suppress EAE [49,50]. GA is a random copolymer that is comprised of four amino acids found in MBP: tyrosine, glutamate, alanine, and lysine [51]. It was predicted that GA would be encephalitogenic given its sequence similarity with MBP, but it is surprisingly immunosuppressive [49,50]. After over 20 years of animal research, a phase III multicenter clinical trial demonstrated that subcutaneous injections of GA reduce relapse rate by 29% in patients with RRMS and improves neurologic disability [52,53]. Exactly how GA improves EAE and MS outcomes is not clear but EAE studies suggest that multiple mechanisms are involved, including suppression of Th1 and Th17 cell-mediated inflammation; induction of regulatory T cell (Treg) differentiation [54,55,56,57] and the suppressor function of the CD11b^+^ Ly6G^−^ monocytes [58]; increasing proliferation, differentiation and survival of oligodendrocyte progenitor cells [59]; and enhancing production of neuroprotection and regeneration factors in the brain [60].

Natalizumab is another MS drug that was discovered through EAE model [61]. Natalizumab is a second-generation drug for RRMS targeting α4-integrin expressed on CD4^+^ T cells, which together with the β1 subunit, form VLA-4. VLA-4 is important for CD4^+^ T cells entering the brain parenchyma from the blood during the initiation of neuroinflammation [62]. Clinical trials showed that natalizumab treatments lead to fewer brain lesions and relapses in patients with MS [63], and reduce the risk of sustained progression of disability to about 40% over two years [64]. Unlike GA, natalizumab is a mechanistically well-defined drug discovered through molecular targeting using EAE model, further emphasizing the importance of this model in translational MS research.

Similar to other disease models, EAE model has its limitations and does not perfectly mirror MS in humans. EAE is induced by active immunization of CNS antigens with CFA or by adoptive transfer of encephalitogenic CD4^+^ T cells to laboratory mice with identical genetic background [65]. MS, on the other hand, is strongly influenced by genetic polymorphisms of individuals and is triggered by ill-defined environmental factors [38]. Therefore, EAE is not a good model for understanding the initiation and prevention of MS. Mechanistically, autoreactive CD4^+^ T cells play a major role in the pathogenesis of EAE, which are predominately found in the CNS lesions [66], and the role of CD8^+^ T cells and B cells are often shadowed. However, these cells significantly contribute to the MS pathology [67]. In addition, inflammation and lesions are predominately found at the spinal cord after EAE induction, and thus disease severity is mainly evaluated by the physical disability of the mice due to muscle paralysis [68]. MS patients have diverse clinical manifestations which is indicative of lesions at both brain parenchyma and spinal cord. Thus, EAE may not be a suitable model to study cognitive impairments caused by lesions in the brain in MS patients.

One of the major concerns of translating new therapeutics from EAE to MS is that the side effects of some drugs, which may be severe and even fatal in humans, are not observed in animal models. This may be due to a number of reasons, including different dosages being administered to animals and humans, severe side effects not being observed in small scale animal studies, or the absence of environmental factors that trigger the side effect in controlled animal facility. A prominent example indicating the severity of side effect are reports of progressive multifocal leukoencephalopathy (PML) in MS patients treated with natalizumab [69], leading to a temporary withdrawal of this drug from the market [70]. PML is a rare disease caused by oligodendroglial infection with opportunistic John Cunningham (JC) virus, which is present in the microflora of ~50% of human populations [71]. Although natalizumab was later re-introduced to the clinic for treating MS patients who do not carry JC virus [72], this example reminds clinicians and scientists that carefully designed clinical trials and post-market evaluations of new MS therapeutics are necessary even when promising results are obtained from EAE model.

Besides EAE model, viral- and toxin-induced demyelination models are also used for MS studies. Infectious agents are possible triggers of MS, which is supported by reports showing that some viruses can cause CNS encephalomyelitis [73]. Theiler’s murine encephalomyelitis virus-induced demyelinating disease (TMEV-IDD) is an example of a viral-induced animal model of MS. TMEV is a single-stranded RNA virus which belongs to cardiovirus genus of *Picornaviridae* family [74,75]. Based on neurovirulence, TMEV is classified into two subgroups. The high virulence group consists of GDVII and FA strains which can cause acute encephalitis and death of animal within 1 to 2 weeks of injection [76,77]. The less virulent group is Theiler’s original (TO) subgroup which includes BeAn 8386 (BeAn) and Daniels (DA) strains, can cause acute encephalitis after a week of injection and a more chronic phase of disease a month after infection [75,76,78]. Similar to EAE, TMEV-induced demyelination is through an immune-mediated mechanism and the role of cytokines in this model will be also discussed briefly.

Cuprizone (bis-cyclohexanone-oxalyldihydrazone, CPZ) and lysolecithin are the most commonly used toxins for inducing demyelination in the animal models of MS [79,80,81]. Toxin-induced animal models of MS provide a better scope for studying the non-immune-mediated demyelination and remyelination events [82]. In CPZ-induced animal model of demyelination, susceptible strains of mice are fed with 0.2% of cuprizone-supplemented diets for 4 weeks, which results in demyelination in various regions of the brain, mainly due to the death of oligodendrocytes [79,80,83]. Withdrawal of CPZ from the diets results in spontaneous remyelination. However, if cuprizone diets are continued for 12 weeks, it results in induction of chronic demyelinating lesions [79,81]. CPZ model involves non-inflammatory-mediated demyelination by inducing metabolic stress in oligodendrocytes. This leads to apoptosis of these cells and myelin destruction, and ultimately causes axonal and neuronal pathology [80,84]. Local injection of lysolecithin into spinal cord of animal results in induction of focal areas of demyelination [79], which is another non-immune-mediated model for studying the process of demyelination and remyelination [85]. Cytokines contributing to the pathogenesis of the toxin-induced demyelination is less clear.

## 5. Cytokines Involved in the Pathogenesis of Multiple Sclerosis and Experimental Autoimmune Encephalomyelitis

Although the precise mechanism for the initiation of MS is still not clear, the pathogenic cascade leading to EAE has been well studied and reviewed in detail [86]. Briefly, autoreactive CD4^+^ T cells are differentiated into pathogenic Th1 and Th17 cells in the secondary lymphoid organs, then egress to the CNS via crossing the choroid plexus. These T cells interact with the APCs residing at the subarachnoid and perivascular space, leading to the expansion of autoreactive T cells, production of pro-inflammatory cytokines, increasing subpial blood vessel permeability, and causing influx of circulating autoreactive T cells and effector myeloid cells. The massive production of pro-inflammatory cytokines by the infiltrated immune cells ultimately destroy BBB integrity, resulting in inflammation and tissue damage [38,78]. Recent transcriptome analysis demonstrated that the myelin-specific CCR6^+^ T cells from MS patients share gene signatures with the EAE-driven pathogenic Th17 cells [23], suggesting that the above paradigm is to some extent applicable to the pathogenesis of MS.

Cytokines are critically involved throughout the course of MS, from the initial pathogenic T-cell differentiation in the periphery, to the resulting inflammation and tissue damage in the CNS (Table 1). Manipulation of cytokine availability and/or signaling is an attractive strategy for MS treatment as this approach is currently used for treating different autoimmune diseases. For example, tumor necrosis factor (TNF)-α inhibitors are used for treating rheumatoid arthritis and Crohn’s disease. Early studies showed that TNF-α level is elevated in the CSF of MS patients, and correlates with both disease severity and progression [87,88]. Moreover, antibody neutralizing TNF-α prevents EAE induced by adoptive transfer of MBP-specific T cell line [89]. Further, increased expression of TNF-α was observed in the CNS of SJL/J mice infected with TMEV [90]. Treatment of TMEV-infected mice with antibody specific for TNF-α at the onset of disease results in suppression of disease development [91]. This suggests that TNF-α inhibitors might also be effective in treating MS. Unfortunately, clinical trials showed that MS patients treated with recombinant TNF receptor lenercept (sTNFR-IgG p55) experienced exacerbated disease pathology [92], indicating TNF-α may have multiple, cell type-specific roles in neuroinflammation, some of which may be neuro-protective [93].

Disease amelioration may also be achieved by elevating the level of cytokines that suppress immune functions. Interferon (IFN)-β1b was the first approved drug for treating RRMS in 1993. At that time, the mechanism in which IFN-β reduces relapse rate was unclear, but it was thought to be related to its anti-viral effects [94,95]. Therefore, the effect of IFN-γ in patients with RRMS was also tested but disease exacerbation was observed [96].

Further studies have shown that IFN-β indeed suppresses neuroinflammation via multiple mechanisms, including the induction of Tregs and suppression of Th17 cell differentiation [97,98]; reduction of the inflammatory cell migration into the CNS [99]; suppression of DC activity [100]; and the induction of IL-10 [101]. The ability of type I interferon to induce IL-10 production is particularly of interest as it has been shown that the IL-10-producing regulatory B cells (B10 cells) significantly reduce EAE severity [102]. ATX-MS-1467 is a mixture of four peptides of human MBP, and injection of these peptides inhibits EAE correlated with IL-10 induction [103]. A phase 2a clinical trial of this agent in RRMS was recently completed (NCT01973491) and results are expected to be released soon.

## 6. The IL-23-IL17 Axis in Multiple Sclerosis and Experimental Autoimmune Encephalomyelitis

Targeting cytokines controlling CD4^+^ T-cell differentiation and effector functions is certainly a rational strategy to ameliorate MS. Prior to the emergence of the concept of Th17 lineage, Th1 cells were the main target. However, several studies have showed that IFN-γ—deficient mice have higher susceptibility to EAE [104,105,106], and IFN-γ unpredictably was found to suppress the expansion of CD4^+^ T cells during the course of EAE [104]. In TMEV model of demyelination, treatment with anti-IFN-γ antibody results in increased disease severity in SJL/J mice [107]. IL-12, composed by the p40 and p35 subunits, is the driver of Th1 differentiation [108,109]. Surprisingly, although mice lacking IL-12p40 are resistant to EAE, mice lacking IL-12p35 are susceptible [110,111], suggesting that the IL-12-Th1 axis may not be a major contributor to the EAE pathology, and IL-12p40 may have a biological function other than dimerizing with IL-12p35. This question was soon resolved by the discovery that the p40 subunit can dimerize with a p19 subunit, and together they form IL-23. Correspondingly, IL-23p19-deficient mice are resistant to EAE. Further studies showed that the IL-23-dependent CD4^+^ T cells are highly pathogenic and are characterized by the production of IL-17, IL-6, and TNF [112]. Later the same year, the concept of a distinct Th17 lineage was proposed [48,113] and there was intense interest in understanding the role of these cells in autoimmunity. In TMEV model, Th17 cells contribute to the viral persistence and chronic demyelination, and IL-17 neutralization increases virus clearance and enhances cytotoxic T cell functions [114]. Th17 cells are characterized by the production of IL-17A, IL-17F, IL-21, and IL-22, and can be differentiated in vitro by the transforming growth factor (TGF)-β and IL-6 or IL-21 without the need of IL-23 [115,116,117,118]. However, adoptive transfer of Th17 cells differentiated by TGF-β1 and IL-6 are unable to induce severe EAE is the absence of IL-23 [119]. Correspondingly, disease severity was only partially reduced when EAE was induced by active immunization in IL-17—deficient mice, or by adoptive transfer of lymphocytes isolated from *Il17a*^−/−^ mice [120,121], suggesting there may be an additional function of IL-23 in neuroinflammation onset. This was shown by studies demonstrating that IL-23 induces the production of granulocyte-macrophage colony-stimulating factor (GM-CSF) in IL-17 cells, and GM-CSF is the main pathogenic factor of EAE [120,122].

## 7. GM-CSF Connects the Priming Autoreactive CD4^+^ T Cells to the Effector Myeloid Cells

GM-CSF is not a new mediator of MS and EAE. McQualter et al. [123] in 2001 reported that *Csf2*^−/−^ mice (which encodes GM-CSF) are resistant to EAE development, correlating with reduced immune cell infiltration into to CNS and diminished proliferation of MOG_35–55_-specific splenic lymphocytes. Intraperitoneal injection of anti-GM-CSF monoclonal antibody (mAb) significantly reduces EAE severity, suggesting it has promising therapeutic potential [123]. In humans, myelin-reactive CD4^+^ T cells isolated from MS patients demonstrate an increase in the production of GM-CSF [23], along with an increase in the frequency of GM-CSF-producing memory Th cells [124,125,126]. Importantly, MS patients treated with various disease-modifying drugs, including IFN-β, natalizumab, and GA, have reduced frequencies of the GM-CSF-producing T cells [125,126], further supporting the rationale of targeting GM-CSF signaling as MS therapy.

**Figure 1:**
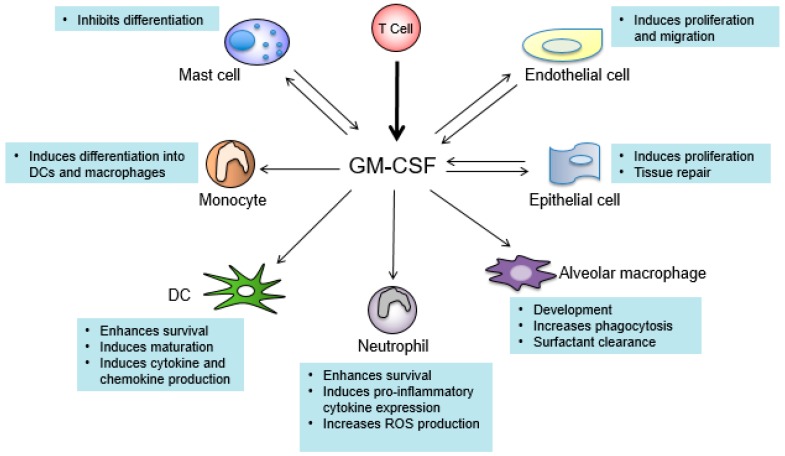
Cell types that produce and respond to granulocyte-macrophage colony-stimulating factor (GM-CSF). Arrows pointing toward GM-CSF indicate cell types that produce GM-CSF. Arrows pointing away from GM-CSF indicate cells that respond to GM-CSF. T cells (bold arrow) are the main producers of GM-CSF. Texts describe the major functions of GM-CSF in the indicated cell types. DC: dendritic cell; ROS: reactive oxygen species.

Understanding the signaling pathways triggered by GM-CSF and its biological functions is critical for designing novel therapeutics with minimal side effects. GM-CSF is a monomeric glycoprotein which was first cloned in mice in 1984 and then in humans in 1985 [127,128]. GM-CSF is primarily produced by T cells, macrophages, mast cells, fibroblasts, and endothelial cells [129]. The GM-CSF receptor (GM-CSFR) is composed by the α chain which is GM-CSF-specific, and the common β chain (β_c_) that is shared with IL-3 and IL-5 [130]. GM-CSFR is expressed in multiple lineages of cells including monocytes, macrophages, neutrophils, DCs, endothelial cells, and others (Figure 1). GM-CSFR is associated with the tyrosine kinase JAK2 [131]. GM-CSF binding leads to autophosphorylation of JAK2, followed by phosphorylation and activation of the downstream signal transducer of activation of transcription (STAT) proteins. STAT5 is the major STAT protein activated by GM-CSF, which drives transcription of genes responsible for immune cell differentiation and the onset of inflammation [132]. GM-CSF also stimulates the activation of nuclear factor kappa-light-chain-enhancer of activated B cells (NF-κβ), mitogen activated protein kinase (MAP kinase), and phosphoinositide 3 kinase (PI3K) pathways [133].

Under non-inflammatory condition, GM-CSF is critical for the differentiation of alveolar macrophages (AMs) via the regulation of transcription factor PU.1 [134,135]. *Csf2*^−/−^ mice showed defects in innate immune functions [135]. Importantly, mutation of GM-CSFRα or the common β chain in humans results in pulmonary alveolar proteinosis (PAP), which is due to defective development of AMs and reduction of surfactant clearance [136,137]. In addition, GM-CSF neutralizing antibodies have been found in the bronchoalveolar lavage fluid of patients with idiopathic PAP [138], suggesting that GM-CSF is essential for maintaining pulmonary innate immunity.

It has been known for some time that GM-CSF can drive the differentiation of bone marrow progenitor cells into dendritic cells and macrophages [139,140]. Monocytes and DC precursors (pre-DCs) in the bone marrow are target progenitor cells of GM-CSF [141]. Recent studies have shown that GM-CSF-stimulated CCR2^+^ monocytes are critical for the pathogenesis of EAE [132,142]. Mice with *Csf2rb* conditionally deleted in Ly6C^hi^ CCR2^+^ monocytes are resistant to the development of EAE, which is phenocopied in the complete *Csf2rb* knockout [132], suggesting that even though GM-CSF can act on many immune cell types, its effects on monocytes are the most critical for driving neuroinflammation. Furthermore, using a transgenic mouse line in which GM-CSF expression can be induced in peripheral CD4^+^ T cells, Spath et al. recently demonstrated that GM-CSF secretion promotes an antigen-independent invasion of inflammatory myeloid cell into the CNS, leading to tissue damage [143]. Myeloid cells invading into the CNS have a distinct genetic profile compared to those invading the lung, liver, and kidney [143]. The mechanism causing this difference remains to be determined.

Much effort has been made in the past few years to understand the mechanisms driving GM-CSF production in Th17 cells and its correlation with autoimmunity. Pertussis toxin-stimulated IL-1β production in myeloid cells promotes the expansion of GM-CSF-producing Th17 cells, which enhance their encephalitogenic potential [144]. IL-1β likely works upstream of IL-23 signaling, as the EAE pathogenicity of IL-1R1-deficient T cells is fully restored by the addition of exogenous IL-23 during in vitro polarization and expansion [144]. In addition, mice lacking protein kinase CK2 show reduced Th17-cell development and attenuated expression of IL-17, GM-CSF, and IL-23 receptor [145]. Pharmacological inhibition of CK2 activity ameliorates EAE severity and relapse incidence [145]. Of note, however, Th lineages other than Th17 also produce GM-CSF. Sheng et al. showed that GM-CSF expression can be induced by IL-7-mediated signaling in CD4^+^ T cells independent of IFN-γ and IL-17 [146]. GM-CSF can also be induced in conventional αβ and γδ T cells under IL-12- or IL-23-stimulated conditions [147]. Surprisingly, GM-CSF expression in human Th cells is induced by IL-12 but is suppressed by IL-23 [148], indicating that there are distinct GM-CSF regulatory pathways in humans and mice. The diverse sources of GM-CSF during inflammation may explain why injections of anti-IL-12p40 mAb (ustekinumab) failed to improve clinical outcomes in patients with RRMS [149]. This result, although disappointing, further supports the notion that GM-CSF, but not IL-17 nor other Th17 cell-producing cytokines, drives autoimmunity in the CNS.

Recently, the first drug for treating progressive MS, ocrelizumab, has been approved by the United States Food and Drug Administration. Ocrelizumab is a humanized anti-CD20 mAb which depletes non-antibody-secreting, CD20-expressing B cells [150]. This positive result suggests that B cells have regulatory role(s) that contributes to the pathogenesis of progressive MS, and that regulatory role may be involved in the production of GM-CSF [151]. Therefore, targeting GM-CSF or its downstream signaling may have potential in treating progressive MS.

## 8. Conclusions

Both clinical data and EAE model have provided strong evidence that targeting GM-CSF or GM-CSFR is a promising strategy for treating MS, and research in this area is underway. A recent study demonstrated that blocking GM-CSF signaling by anti-GM-CSFRα antibody results in amelioration of EAE progression [152]. In addition, a randomized phase 1b trial of anti-GMCSF mAb (MOR103) in patients with RRMS or SPMS has proven its tolerability [153] and therefore warrants further efficacy studies. However, although immunotherapy utilizing monoclonal antibodies has been proven successful in many disease settings, antibodies targeting GM-CSF or GM-CSFR have to be used with caution. As discussed above, GM-CSF is critical for the terminal differentiation of AMs via PU.1 [135], and GM-CSF autoantibodies are detected in patients with PAP [138]. Complete inhibition of GM-CSF signaling therefore may lead to lung pathology. Thus, mechanistic studies are needed to further distinguish signaling pathways responsible for driving immune cell development and functions under physiological versus pathological conditions, so that partial ablation of GM-CSF activity by targeting its downstream signaling molecules, such as the PU.1-independent pathway, may be feasible for achieving beneficial clinical outcomes.

## Figures and Tables

**Table 1:** Major cytokines contributing to the pathogenesis of multiple sclerosis (MS) and experimental autoimmune encephalomyelitis (EAE).

Cytokine	Main Producers	Levels in MS Patients	Role in EAE	Potential Treatments of MS
GM-CSF	T cells	Elevated	GM-CSF-deficient mice are completely resistant to EAE [123]	Phase 1b trial of humanized anti-GM-CSF mAb MOR103 in MS is completed [153]
IFN-β	pDCs	Not reported	*Ifnb*^−/−^ mice exhibit increased EAE severity [154]	First line treatment of RRMS [155]
IFN-γ	Th1 cells, NK cells, NKT cells	Elevated	*Ifng*^−/−^ mice exhibit increased EAE severity [105]	Intravenous infusion of IFN-γ exacerbates disease in MS patients [96]
IL-1β	Monocytes, macrophages	Elevated	*Il1r1*^−/−^ mice are resistant to EAE [156]	Not reported
IL-10	Tregs, macrophages, DCs, B cells	Reduced	*Il10*^−/−^ mice exhibit increased EAE severity [157]	Not reported
IL-12	DCs, macrophages	Elevated	IL-12 *p35^−/−^* exhibit increased EAE severity [110]	Anti-IL-12/IL-23 p40 mAb Ustekinumab does not show efficacy in treating RRMS in phase II trial [149]
IL-17	Th17 cells, γδ T cells, NKT cells	Elevated	*Il17a*^−/−^ mice are partially resistant to EAE [121]	Anti-17A mAb Secukinumab reduces disease severity in RRMS patients [158]
IL-23	DCs, macrophages	Elevated	*Il23r*^−/−^ mice are completely resistant to EAE [47]	Anti-IL-12/IL-23 p40 mAb Ustekinumab does not show efficacy in treating RRMS in phase II trial [149]
TNF-α	Macrophages	Elevated	*Tnfrsf1a*^−/−^ mice are partially resistant to EAE [159]	Treatment of MS patients with anti-TNF-α exacerbates disease in MS patients [160]

GM-CSF: granulocyte-macrophage colony-stimulating factor; IFN: interferon; IL: interleukin; TNF: tumor necrosis factor; DCs: dendritic cells; pDCs: plasmacytoid dendritic cells; NK: natural killer; NKT: nature killer T; Tregs: regulatory T cells; RRMS: relapsing-remitting multiple sclerosis; mAb: monoclonal antibody.
